# *Arabidopsis* Seed Content QTL Mapping Using High-Throughput Phenotyping: The Assets of Near Infrared Spectroscopy

**DOI:** 10.3389/fpls.2016.01682

**Published:** 2016-11-10

**Authors:** Sophie Jasinski, Alain Lécureuil, Monique Durandet, Patrick Bernard-Moulin, Philippe Guerche

**Affiliations:** ^1^Institut Jean-Pierre Bourgin, INRA, AgroParisTech, CNRS, Université Paris-SaclayVersailles, France; ^2^ThermoFisher ScientificCourtaboeuf, France

**Keywords:** *Arabidopsis thaliana*, seed storage contents, near infrared spectroscopy, plant, natural variation, quantitative trait loci

## Abstract

Seed storage compounds are of crucial importance for human diet, feed and industrial uses. In oleo-proteaginous species like rapeseed, seed oil and protein are the qualitative determinants that conferred economic value to the harvested seed. To date, although the biosynthesis pathways of oil and storage protein are rather well-known, the factors that determine how these types of reserves are partitioned in seeds have to be identified. With the aim of implementing a quantitative genetics approach, requiring phenotyping of 100s of plants, our first objective was to establish near-infrared reflectance spectroscopic (NIRS) predictive equations in order to estimate oil, protein, carbon, and nitrogen content in *Arabidopsis* seed with high-throughput level. Our results demonstrated that NIRS is a powerful non-destructive, high-throughput method to assess the content of these four major components studied in *Arabidopsis* seed. With this tool in hand, we analyzed *Arabidopsis* natural variation for these four components and illustrated that they all displayed a wide range of variation. Finally, NIRS was used in order to map QTL for these four traits using seeds from the *Arabidopsis thaliana* Ct-1 × Col-0 recombinant inbred line population. Some QTL co-localized with QTL previously identified, but others mapped to chromosomal regions never identified so far for such traits. This paper illustrates the usefulness of NIRS predictive equations to perform accurate high-throughput phenotyping of *Arabidopsis* seed content, opening new perspectives in gene identification following QTL mapping and genome wide association studies.

## Background

Plant seeds constitute a key component of both human and livestock diets, as seed storage compounds are mainly composed of protein, oil and starch. Seed oil from oleaginous crops are composed mainly of triacylglycerols, which are structurally similar to long chain hydrocarbons derived from petroleum, and thus represent ecologically and economically competitive alternatives to petroleum-based products for the production of molecules for green chemistry (e.g., detergents, paints, plastics, and lubricants) as well as for the production of biofuels (Durrett et al., 2008; Dyer et al., 2008). The increasing demand of plant-derived products for nutritional and industrial applications highlights the urgent need to develop new methodologies to increase the overall seed oil and protein content. Although, most of the biochemical steps involved in oil and protein biosynthesis are known and the key genes have been identified (Shewry et al., 1995; Beisson et al., 2003), the regulation of the processes that results in the final oil and protein content is not well-understood. Even more, the genetic factors that control the oil/protein ratio in the seeds have to be identified.

Seed oil and protein accumulation processes, like many important agronomical traits are quantitative and have a complex genetic basis. The method most commonly used for inferring the presence and position of such genes in the genome is based upon analysis such as QTL and more recently genome wide association studies (GWASs). Quantitative genetics has been used to search for genetic factors controlling oil and/or protein quantity in a variety of agronomically important species including rapeseed (Sun et al., 2012; Li et al., 2014), soybean (Eskandari et al., 2012; Hwang et al., 2014), maize (Zheng et al., 2008; Li et al., 2012; Yang et al., 2012), pea (Irzykowska and Wolko, 2004; Tar’an et al., 2004), rice (Ying et al., 2012), wheat (Plessis et al., 2013), oat (Kianian et al., 1999), sunflower (Mokrani et al., 2002), linseed (Kumar et al., 2015), cotton (Liu et al., 2015), and Jatropha (Liu et al., 2011). However, with the exception of one QTL affecting seed oil content in maize (Zheng et al., 2008), none of these studies have gone beyond the gene mapping stage. In the model species *Arabidopsis*, using natural variation resources, QTL involved in seed oil content and/or quality have also been detected (Hobbs et al., 2004; Jasinski et al., 2012; O’Neill et al., 2012; Sanyal and Linder, 2012) as well as “regions of interest” by GWAS (Branham et al., 2015), but only the study published by Jasinski et al. (2012) identified the gene involved. Since QTL cloning is often easier in model species for which substantial genetic resources exist, we implemented a QTL approach to study storage compound metabolisms in *Arabidopsis* seed (Jasinski et al., 2012; Chardon et al., 2014). Seed metabolism is very similar between *Arabidopsis* and *Brassica* species and the close relationship between them allows the use of comparative genetics to predict orthologous genes and alleles within the *Brassica* genome (Parkin et al., 2005). This will enable the translation of discoveries from *Arabidopsis* into Brassicaceae and other crops breeding programs.

Quantitative genetics relies on statistical links between phenotype and genotype, implying genotyping and phenotyping of 1000s of lines. In *Arabidopsis*, genotyping is not a limiting factor and many genetic and genomic resources are available including complete genome sequence of many accessions, data on gene structure, gene expression, DNA and seed stocks, genome maps, molecular markers. Seed oil and protein content are usually determined by standard analytical methods such as Soxhlet or gas chromatography (following fatty acid methyl ester extraction) for oil content and combustion analysis or Kjeldahl for protein content. Although, these standard analytical techniques offer a high level of accuracy and precision, they also show some limitations, such as indirect determination (combustion analysis and Kjeldahl determine nitrogen content rather than actual protein content), high costs, time-consuming experiments and use of hazardous chemicals. For many of these reasons, they are not fully appropriate for high-throughput phenotyping required in genetics approaches. Near infrared spectroscopy (NIRS) is a vibrational spectroscopy technique, providing a spectrum representative of the “signature” of all components present in the analyzed sample. It possesses numerous advantages compared to classical analytical techniques. NIRS analyses show high degree of repeatability and are carried out with considerable saving of time (spectrum acquisition lasts only a few seconds), cost and without using hazardous chemicals. In addition, samples can be analyzed in their natural form without destruction neither any special sample preparation. However, a calibration has first to be established: regression modeling is used to relate NIRS spectra to chemical concentrations determined by a standard analytical method. After calibration, the developed regression equations allow accurate analysis of many other samples by prediction of data based on the spectra. Moreover, from only one spectrum, different components can be predicted using different predictive equations. In recent decades, NIRS has been widely used as a fast and reliable method for qualitative and quantitative analysis in many fields (Font et al., 2006) and International Standards Committees have formally accepted methods using NIRS for analysis of many compounds (Batten, 1998). Regarding *Brassica* seeds, many authors have reported NIRS models for different components, such as glucosinolates (Velasco and Becker, 1998; Font et al., 2004), fiber (Font et al., 2003), protein and oil contents (Tkachuk, 1981; Font et al., 2002a,b; Rossato et al., 2013).

Surprisingly, NIRS technique has not been applied to the analysis of *Arabidopsis* seed. Some people used nuclear magnetic resonance spectroscopy (NMR) as rapid technique to measure *Arabidopsis* seed oil content (O’Neill et al., 2003, 2012; Hobbs et al., 2004). However, NMR is not suitable for protein detection and was thus not suitable for our purpose. In this study, the potential of NIRS was evaluated for the simultaneous analysis of total oil and protein content of *Arabidopsis* seeds, as well as nitrogen and carbon contents, which allow studies of global metabolic fluxes. A calibration set of 90-112 seed samples was subjected to both NIRS and appropriate reference methods and predictive equations for seed (1) oil, (2) protein, (3) carbon, and (4) nitrogen content were developed.

These equations were further used to analyze *Arabidopsis* natural variation for these four major seed components. Finally, a search for genetic factors governing the accumulation of these four components in *Arabidopsis* seed was carried out by a QTL analysis (this work and Chardon et al., 2014), allowing the mapping of new QTL involved in seed oil and protein content.

## Results

### Development of NIRS Predictive Equations for Seed Oil, Protein, Carbon, and Nitrogen Content

Four calibration models in order to predict oil, protein, carbon, and nitrogen content in *Arabidopsis* seeds were developed as indicated in Section “Methods.”

**FIGURE 1:**
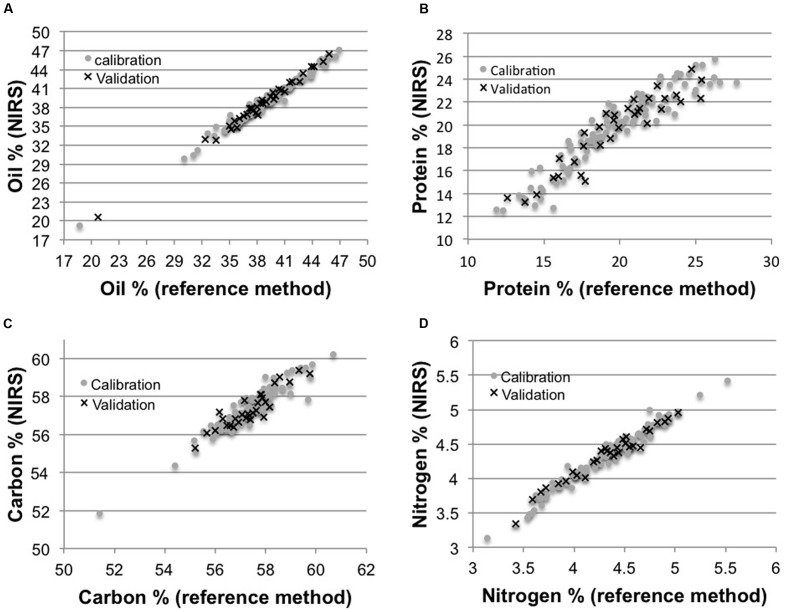
**Regression curves between near-infrared reflectance spectroscopic (NIRS) and reference methods for oil, protein, carbon, and nitrogen content.** Scatter plots of NIRS versus reference method values for oil % **(A)**, protein % **(B)**, carbon **(C)**, and nitrogen **(D)** in the calibration and validation sets.

The oil calibration set of 112 samples showed a wide range of variation for oil content from 18.70%, corresponding to the *wri1* low-seed-oil T-DNA insertion mutant (Focks and Benning, 1998), to 46.90%, with a mean of 38.78% (**Table 1**) and a standard deviation (SD) of 3.88%. The predictive equation for seed oil content was developed with five partial least square (PLS) factors and first evaluated through cross-validation (leave-one-out method). Very high coefficient of determinations between Soxhlet and NIRS values were observed for both calibration and cross-validation ($r_{C}^{2}$ = $r_{CV}^{2}$ = 0.98, **Table 1**; **Figure 1A**). The standard error of cross-validation (SECV) was 0.606% (**Table 1**). The 36 additional seed samples were used to carry out an external validation to better assess the accuracy of this calibration model. This showed a coefficient of determination of 0.99 and a standard error of prediction (SEP) of 0.505% (**Table 1**; **Figure 1A**).

**Table 1:** Near-infrared reflectance spectroscopic (NIRS) calibration and cross validation statistics for seed oil, protein, carbon, and nitrogen contents (%).

	Calibration						Cross-validation		External validation					
	
	*n*	Mean (%)	Range (%)	*SD*	PLS factors	SEC	$r_{C}^{2}$	SECV	$r_{CV}^{2}$	*n*	Mean (%)	*SD*	SEP	$r_{V}^{2}$	RPD
Oil	112	38.78	18.70–46.90	3.88	5	0.541	0.98	0.606	0.98	36	38.45	4.39	0.505	0.99	7.68
Protein	98	19.99	11.89–27.73	3.39	3	1.219	0.88	1.287	0.85	33	19.73	3.32	1.228	0.86	2.76
Carbon	91	57.39	51.41–60.69	1.25	3	0.424	0.88	0.460	0.86	30	57.39	1.04	0.432	0.82	2.90
Nitrogen	90	4.34	3.14–5.52	0.43	4	0.081	0.96	0.091	0.95	30	4.32	0.41	0.087	0.96	4.91


**n*, number of samples; *SD*, standard deviation; PLS, partial least square; SEC, standard error of calibration; $r_{C}^{2}$, coefficient of determination in calibration; SECV, standard error of cross-validation; $r_{CV}^{2}$, coefficient of determination in cross-validation; $r_{V}^{2}$, coefficient of determination in validation; SEP, standard error of prediction; RPD, ratio of performance deviation (SD/SEP).*

As for oil, the three other calibration sets showed a wide range of variation (**Table 1**). For each of the three components, a predictive equation was developed and the external validation sample set was further used to evaluate its performance. For the three developed models, a limited number of PLS factors (≤4) was used and very high coefficients of determination between the reference method and NIRS values were observed for both calibration and cross-validation (**Table 1**; **Figure 1**).

The ratio of performance deviation (RPD), an indicator for the usefulness of the calibration model was calculated for each component (**Table 1**). According to the American Association of Cereal Chemists Method-39-00.01 (AACC International, 1999), a RPD ≥ 2.5 indicates that a calibration equation is useful for screening in breeding programs, a RPD ≥ 5 that a calibration is acceptable for quality control and a RPD ≥ 8 that a calibration is good for process control, development, and applied research. For all the models developed in this study, a RPD > 2.5 was achieved, with a RPD of 7.68 (close to 8) for the oil content model and a RPD close to 5 for the nitrogen model. This indicates that the four models developed in this study are suitable for quantitative genetic approaches.

### NIRS Is a Suitable Tool for High-Throughput Phenotyping of *Arabidopsis* Seed

**FIGURE 2:**
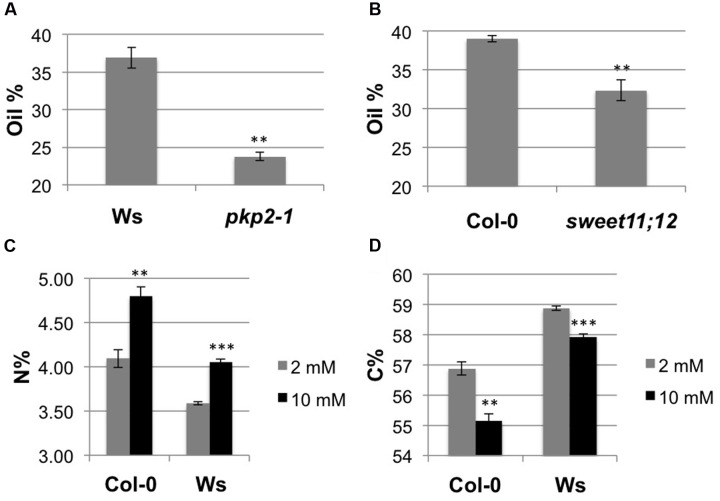
**Near-infrared reflectance spectroscopic phenotyping allows detecting seed filling modifications.** **(A,B)** Graphs showing seed oil content of two mutants: *pkp2-1* **(A)** and *sweet11;12* **(B)** compared to wild-type. **(C,D)** Graphs showing seed nitrogen **(C)** and carbon **(D)** content of two accessions (Col-0 and Ws) on two nitrogen nutrition conditions (2 and 10 mM). Bars represent SE (*n* = 3 in **A,B**, *n* = 4 in **C,D**). Significance in *t*-test, ^∗∗^*p* < 0.01, ^∗∗∗^*p* < 0.001.

In order to fully demonstrate that the developed NIRS models were suitable to study seed composition in *Arabidopsis*, we analyzed mutants altered in seed filling. Pyruvate kinase (PK) catalyze the irreversible synthesis of pyruvate and ATP (Valentini et al., 2000), which are essential for fatty acid production in the plastids of maturing *Arabidopsis* embryos. Baud et al. have shown that the plastidial PK isoform PKp2 plays an important role in seed oil synthesis, with *pkp2-1* mutant exhibiting a 50% reduction in seed oil content compared to wild-type (Baud et al., 2007). More recently, Chen et al. showed that seed filling in *Arabidopsis* requires sucrose transporters from the SWEET family (Chen et al., 2015). In particular, they showed that seed oil content was reduced by 34% in the *sweet11;12* double mutant (Chen et al., 2015). Seeds from *pkp2-1* and *sweet11;12* mutants were analyzed by NIRS. For *pkp2-1*, a 36% decrease in oil content compared to wild-type was observed (**Figure 2A**), which is comparable to the decrease described by Baud et al. (2007) on the same seed lot. S*weet11;12* mutants displayed a 17% reduction in seed oil content compared to wild-type (**Figure 2B**), which is half the one described by Chen et al. (2015) on another seed lot. The *sweet11;12* seed lot measured by NIRS was then subjected to Gas Chromatography and resulted in a 15% decrease in seed oil (result not shown). This result suggested that the difference observed is probably due to environmental effect on seed filling more than to NIRS method.

Furthermore, since it is known that nitrogen nutrition impacts seed filling (Masclaux-Daubresse and Chardon, 2011), we analyzed seed content of wild-type plants (Col-0 and Ws) grown under low nitrogen (LowN; 2 mM nitrate) or under high nitrogen (HighN; 10 mM nitrate) nutrition conditions. As already published (Masclaux-Daubresse and Chardon, 2011), both accessions displayed higher seed nitrogen content under HighN compared to LowN (**Figure 2C**) and reversely a higher seed carbon content under LowN compared to HighN (**Figure 2D**).

These results demonstrate that NIRS is a powerful method to determine *Arabidopsis* seed composition.

### Natural Variation for Oil, Protein, Carbon, and Nitrogen Content in *Arabidopsis* Seed

**FIGURE 3:**
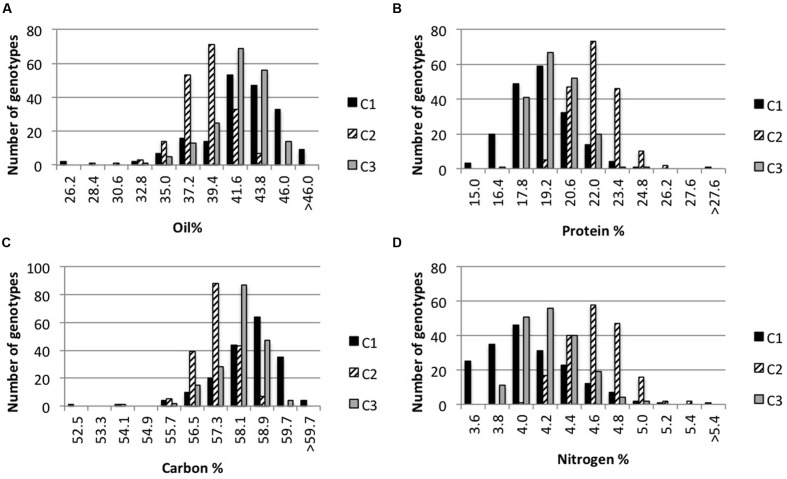
**Frequency distribution of seed oil, protein, carbon, and nitrogen content in three independent cultures.** Frequency distributions of oil **(A)**, protein **(B)**, carbon **(C)**, and nitrogen **(D)** content mean values for 183 genotypes (*n* = 3) corresponding to *Arabidopsis* accessions and RILs from eight populations. The same genotypes were cultivated in three independent cultures (C1, C2, C3).

The development of NIRS predictive equations allowing high-throughput phenotyping opened the door to quantitative genetics study. First, we decided to explore *Arabidopsis* natural variability for oil, protein, carbon, and nitrogen content in seeds. For this purpose, we cultivated the Versailles BRC 48 core-collection of *Arabidopsis* in addition to the Col-0 accession and mini sets of 20 lines (maximizing genotypic variability, Simon et al., 2008) from eight populations (see Methods). Each genotype was cultivated in triplicate and three successive and independent cultures (C1, C2, and C3) were performed in growth chambers with similar global climate conditions. We estimated the natural variability of the four traits by NIRS phenotyping (**Figure 3**). The four traits displayed a wide range of variation in each culture, with C1 displaying the wider range going from 23.23 to 47.72% for oil, from 14.24 to 28.72% for protein, from 52.27 to 60.22% for carbon, and from 3.27 to 5.78% for nitrogen. The modal class is different in each culture, highlighting the environmental effect on these four traits.

**FIGURE 4:**
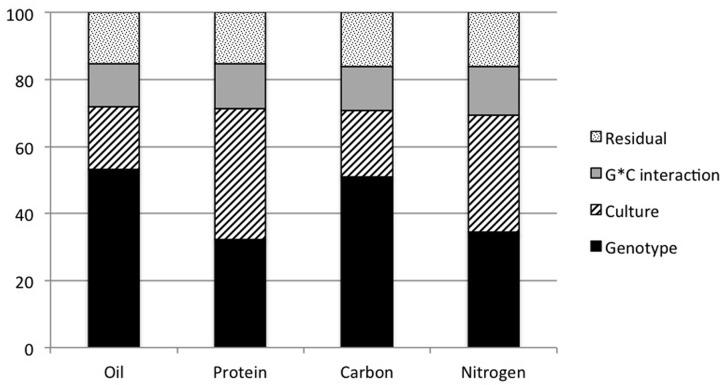
**Variance component analysis for seed oil, protein, carbon, and nitrogen content.** An ANOVA was performed on all genotypes from the three cultures for seed oil, protein, carbon, and nitrogen content. Histograms show the effects due to genotype, culture, interaction genotype^∗^culture and the residual as a percentage of the variation explained.

In order to quantify the relative contribution of the genotype (G), the culture (C) and the G*C interaction on the variation of these four traits, a global analysis of variance (ANOVA) was performed on the measures from the three cultures (**Figure 4**). The genotypic effects ranged from 53.0% for oil to 32.3% for protein, explaining the most important part of the total phenotypic variation except for protein. However, culture effect explained an important part of the total phenotypic variation varying from 19 to 39% for oil and protein respectively. This result showed that nitrogen and protein contents were more influenced by the culture than oil and carbon.

### Identification of QTL Involved in Seed Oil and Protein Content

The availability of NIRS predictive equations together with the large range of variation observed and the high contribution of genetic part to phenotypic variation for oil, protein, carbon, and nitrogen content opened the door to QTL study. From this previous study of natural variation, the Ct-1 × Col-0 RIL population was selected for QTL determination. A subset of 164 RILs, optimized for QTL mapping (Simon et al., 2008) was cultivated (see Methods) and seeds were phenotyped for oil, protein, carbon, and nitrogen content by NIRS. These RILs exhibited a wide range of values for these four traits as well as transgression beyond the parental line values (not shown), highlighting the potential of this subset to study the variation of these traits. QTL detection using standard procedures (see Methods) was carried out, allowing QTL detection for oil and protein content (this work) as well as for carbon, and nitrogen content (Chardon et al., 2014).

Five QTL for seed oil content were identified, explaining 44.5% of the total phenotypic variance observed (**Table 2**). The strongest QTL (Oil.4, explaining more than 15% of the phenotypic variance) is located between 31.6 and 46 cM on chromosome 4. Four QTL were detected for protein content, explaining 34% of the total phenotypic variance observed for this trait (**Table 2**). The strongest QTL (Prot.3, explaining more than 10% of the phenotypic variance) co-localized with Oil.4 on chromosome 4. Three out of five oil QTL, Oil.2, Oil.4, and Oil.5 overlapped with protein QTL Prot.1, Prot.3, and Prot4 respectively, but having an opposite effect on the corresponding traits, highlighting the strong negative correlation observed between oil and protein seed content, as already observed for carbon and nitrogen by Chardon et al. (2014). Interestingly, QTL specific to oil (Oil.1, i.e., without noticeable effect on protein) and protein (Prot.2, i.e., without noticeable effect on oil) were also identified in this study. Altogether, these results illustrate that NIRS phenotyping of mature seeds allow identification of genetic factors involved in different pathways of oil and protein accumulation.

**Table 2:** List of QTL detected for oil and protein in the Ct-1 × Col-0 RIL population.

QTL		Position	LOD		Additive	
name	Chr.	(cM)	score	CI (cM)	effect	R^2^ (%)
Oil.1	2	66.9	3.47	47.7–70.8	0.8	4.93
Oil.2	3	6.0	4.57	0–15.8	-0.95	4.73
Oil.3	3	63.6	7.65	48.9–70.9	1.28	9.86
Oil.4	4	36.7	15.30	31.6–46	1.61	15.65
Oil.5	5	13.3	5.58	8.9–17.4	1.82	9.38
Prot.1	3	6.0	4.62	0–11.8	0.58	9.29
Prot.2	4	4.1	4.60	0–12.4	0.61	9.25
Prot.3	4	36.7	5.34	31.6–46	-0.65	10.23
Prot.4	5	13.3	3.84	4.5–17.4	-0.74	5.23


*For each QTL, the chromosome (Chr.), the position of the nearest marker of the LOD score peak with the LOD score at the corresponding marker as well as the confidence interval (CI) are indicated. The additive effect represents the mean effect on trait of the replacement of both Col-0 alleles by Ct-1 alleles at the QTL. *R*^*2*^ represents the proportion of phenotypic variance of the trait explained by the QTL. cM, centiMorgans.*

## Discussion

Quantitative genetic relies on statistical links between phenotype and genotype of 100s of lines. In *Arabidopsis*, genotyping is no more a limiting factor, whereas high-throughput phenotyping can be an obstacle. Thus, our first objective was to establish near-infrared reflectance spectroscopic (NIRS) predictive equations in order to estimate oil, protein, carbon, and nitrogen content in *Arabidopsis* seed with high-throughput level.

Near-infrared reflectance spectroscopic calibration models for these four components were established on entire seeds using PLSs regression and leave-one-out cross-validation technique. To assess the accuracy of each model, an external validation with samples not included in the initial model was carried out.

The four developed models display good performances as evaluated by different parameters of the external validation set such as $r_{V}^{2}$, coefficient of determination; SEP and RPD (**Table 1**). The $r_{V}^{2}$ values range from 0.82 for carbon content to 0.99 for oil content, indicating that the four models developed in this paper show good to excellent quantitative information (Font et al., 2006). As expected, SECV ≥ SEC (and then $R_{CV}^{2}$ ≤ $R_{C}^{2}$) for the four models and SEP ≥ SEC (and then $R_{V}^{2}$≤ $R_{C}^{2}$) for three out of the four models (**Table 1**). SEP < SEC (and $R_{V}^{2}$ > $R_{C}^{2}$) for the oil model, which is unexpected and illustrates that the restricted validation set (36 samples, i.e., about one third of calibration sample number) fits better to the model. For each model, the validation set shows statistics very close to the calibration set, illustrating robustness and absence of overfitting of the models. Concerning oil content, the model described in this paper for *Arabidopsis* display better performance than the ones described for rapeseed (Tkachuk, 1981; Hom et al., 2007; Rossato et al., 2013). Interestingly, the models were developed on entire seeds without destruction neither any special sample preparation, which is a great advantage compared to calibration developed on powder or oil for example (Khamchum et al., 2013) as it’s faster and allow the seeds to be used for other applications.

Seeds produced by a plant are heterogeneous (in size and composition) depending of their position on the mother plant and the environmental conditions during their development. This could induce huge phenotypic variation when phenotyping is performed on very little amount of seeds. The protocol described in this paper overcomes this problem since spectra are determined on a large number of seeds (160 mg, i.e., about 8000 seeds), allowing robust sampling. In favorable environmental condition, an *Arabidopsis* plant produces on average 1 g of seeds, highlighting that the quantity required for NIRS analysis is not a limiting factor. However, in stressful conditions, *Arabidopsis* may produce very few seeds. In this case, NIRS will not be suitable for seed content analysis.

Using *pkp2* and *sweet11;12* described mutants and two different nitrogen nutritions, we demonstrated that NIRS is a powerful method to determine *Arabidopsis* seed composition and that NIRS can probably replace labor intensive methods such as fatty acyl methyl ester extraction followed by Gas chromatography analysis for lipids or elemental analyzer measurements for nitrogen and carbon content.

With NIRS calibrations in hand, natural variation of *Arabidopsis* seed composition was explored. Three independent cultures (C1, C2, and C3) of a 48 core-collection, Col-0 and minimal sets of eight RIL populations were performed, allowing the estimation of environmental effect on the four seed traits analyzed (seed lipid, protein, carbon, and nitrogen content). As shown in **Figures 3** and **4**, the four traits display a wide range of variation and are strongly impacted by the environment. However, most of the genotypes (75, 97, and 91% from C1, C2, and C3 respectively) display oil content between 32.8 and 43.8%, as already observed by O’Neill et al. (2003) while studying 360 accessions. Similarly, in our three experiments, Cvi-0 was recorded with low oil content (36.21, 33.56, and 34.90%) while Ct-1 was recorded with high oil content (46.26, 42.40, and 45.3%) as in O’Neill et al. (2003). Even thought seed composition is strongly impacted by environmental conditions, the four traits analyzed are also controlled by genetic factor as illustrated by **Figure 4**. Indeed nine QTL were identified for seed oil and protein content in the Ct-1 × Col-0 RIL population (**Table 2**). Most of the QTL for oil content co-localized with QTL for protein content but with opposite effect on each traits, highlighting the negative correlation between seed oil and protein content. Oil.1 and Oil.2 co-localized with seed oil content QTL previously identified by Hobbs et al. (2004) in the Ler × Cvi-0 RIL population and by O’Neill et al. (2012) in the Cvi-0 × Ag-0 RIL population for Oil.1. Interestingly, Oil.1 does not co-localize with seed protein QTL in the Ct-1 × Col-0 RIL population, suggesting that Oil.1 may regulate oil content without affecting protein content and thus represents a very good candidate to specifically modify oil content without affecting protein content in *Arabidopsis* seed. Conversely, Prot.2 may regulate protein content independently of oil content and could be used to solely modify seed protein content. Fine mapping is required to confirm Oil.1 and Prot.2 specificity as well as to identify the genes under the nine QTL identified.

## Conclusion

In summary, the results of the present work show that NIRS predictive equations developed in this study can be used to reliably predict oil, protein, nitrogen and carbon content of *Arabidopsis* seed samples without destruction neither any special sample preparation. This high-throughput method opens the way for quantitative genetic such as QTL cloning (up to gene identification and not only detection), as well as GWASs but also to mutant library screening. As a first attempt to identify genetic factor controlling seed oil and protein content, QTL for these traits have been mapped in the *Arabidopsis* Ct-1 × Col-0 RIL population. Some of the oil content QTL detected co-localized with QTL identified previously, thus validated our approach, but many novel QTL were also identified. In particular, to our knowledge, this is the first report of seed protein content QTL in *Arabidopsis*. The fine mapping of some of these QTL is underway and should give new insights on the regulatory pathway involved in *Arabidopsis* seed oil and protein accumulation.

## Methods

### Plant Material

The 48 core-collection of *Arabidopsis* (McKhann et al., 2004) in addition to Col-0 accession, *wri1-3. wri1-4. tag1-2. pkp2-1*, and *sweet11;12* mutants, a minimal set (20 lines) of eight RIL populations (2RV, 3RV, 7RV, 8RV, 13RV, 17RV, 20RV, and 21RV) as well as the core-pop of 164 RILs of the Ct-1 × Col-0 population were used in this study (Simon et al., 2008). *wri1-3. wri1-4. pkp2-1*, and *tag1-2* seeds were provided by S. Baud and *sweet11;12* seeds were provided by R. Le Hir. The other seeds were obtained from the Versailles Biological Resource Centre for *Arabidopsis*^1^. Seeds were sown on damp Whatman filters, stratified for 3 days at 4°C and then transferred to a growth cabinet under long-day conditions at 21°C for 2 days. Three seedlings (with emerging radicle) per genotype were planted in soil in 7 cm pots and transferred to a non-heated and naturally lit greenhouse to be vernalized from November to February. After 8 weeks, one plantlet per pot was randomly retained without phenotype selection. After 12 weeks of vernalization, plants were transferred to a growth chamber under long-day conditions (16/8 h photoperiod at 150 mmol photons m^-2^ s^-1^); 21°C day temperature and 18°C night temperature; relative humidity of 65%. From this time, three times a week the plant trays were moved around the growth chamber to reduce position effects. Bags were put over the plants to prevent seed dispersion as soon as the first silique had turned yellow. The plants were no longer watered once the youngest silique had turned yellow. Plants were kept in the growth chamber until dry and then harvested.

Three cultures (C1, C2, and C3) including the 48 core-collection, Col-0, the minimal sets of the 8 RIL populations and *wri1-3. wri1-4*, and *tag1-2* mutants for C3, as well as one culture (C4) of the core-pop of 164 RILs of the Ct-1 × Col-0 population were performed following this protocol.

### Near-Infrared Spectroscopy

#### NIR Spectra Acquisition

Seed samples were placed in a 9 mm diameter clear glass bottle (Agilent, 5182-0714) on 4 mm height for NIRS spectra acquisition and were analyzed as intact (without any treatment). This corresponds to about 300 μl of *Arabidopsis* seeds (about 160 mg or 8 000 seeds).

Spectra acquisition was performed with a Fourier transform near-infrared (FTNIR) analyzer (Antaris II spectrometer; Thermofisher Scientific, France). Spectra were collected in reflectance mode with an 8 cm^-1^ optical resolution and were obtained as an average of 16 scans. Spectra were collected over the range 4000 to 10000^-1^ and calibrations done using four spectral ranges: from 4100 to 4940 cm^-1^; from 5390 to 6690 cm^-1^; from 6900 to 7130 cm^-1^, and from 7185 to 9000 cm^-1^. These spectral regions provide useful information about the organic signature of the *Arabidopsis* samples and exclude the water spectral regions. They have been selected by looking at the regression vector from the PLS (see Development of NIRS Calibration Models) and using a Thermo proprietary pure component algorithm.

#### Selecting the Samples for NIRS Calibration

The robustness and accuracy of a NIRS model are strongly dependent on the accuracy of the reference method but also of the samples chosen for calibration development. Indeed, the calibration samples have to be representative of the spectral variability and must cover the range of the component concentration of the samples that will be further monitored.

As the NIR spectral variability of *Arabidopsis* seeds was not known, NIR spectra of 650 samples (one spectrum per sample) from two independent cultures (C1 and C2) were collected. Spectra were treated with a multiplicative signal correction (MSC) to correct multiplicative effects due to light scattering in spectral data and a Principal component analysis (PCA) was performed in order to select samples maximizing spectral variability. PC1 and PC2 explained 84.5 and 11.9% of the spectral variation respectively. Their graphic representations were similar to a seed spectrum and suggested that they reflect variations due to differences in spectra baseline or particle size for example. PC3 explained 1.9% of the spectral variation and its graphic representation displayed peaks at wavelengths specific to seed storage compounds. Thus the PC1/PC3 graph was used as a criterion for selecting 100 samples in the population as being more variable on the basis of spectra features (Shenk and Westerhaus, 1991).

Seed oil content of these 100 samples was determined by the Soxhlet reference method and a preliminary calibration model was set up. Using this model, seed oil content of samples available at this time (1788 samples from three independent cultures and including *wri1* and *tag1* low-seed-oil insertion mutants) was predicted. This prediction allowed the selection of 48 additional samples with extreme values (maximal and minimal) in order to extend the range of concentration of the final calibration set. Seed oil content was measured with the Soxhlet method on these 48 additional samples. The same procedure was applied to choose samples for seed protein, carbon, and nitrogen content calibration models.

#### Development of NIRS Calibration Models

Calibration models were developed using TQ Analyst software (Thermofisher Scientific, France) using PLSs regression and leave-one-out cross-validation technique. Prior to the PLS regression, all spectra were pre-treated with the scatter correction MSC and by applying a first derivative transformation and a Norris derivative filter (segment length 5, gap size: 5). The use of derivative spectra instead of the raw optical data to perform calibration is a way of solving problems associated with offsets and overlapping peaks.

Near-infrared reflectance spectroscopic calibration models were established for oil, protein, carbon, and nitrogen content by using a number of PLS factors optimal for each component (i.e., only the primary, most important factors were used, the “noise” being encapsulated in the less important factors). The optimal number of PLS factors was determined as the minimum of the PRESS (predicted residual error sum of squares) curve when doing a leave-one-out cross validation method.

The quality of each calibration model was then evaluated by several parameters: the determination coefficients between concentrations predicted from NIRS and from reference analysis, $r_{c}^{2}$ and $r_{CV}^{2}$, calculated for calibration and cross-validation (leave-one-out) data processing respectively, and their respective standard errors [calibration (SEC) and cross-validation (SECV)].

To assess the accuracy of each newly developed calibration model, an external validation with samples not included in the initial model was carried out. The total number of samples was divided into calibration and external validation sets in a rate 3:1. For that purpose, the samples were ranked according to their reference values and then about one sample every four was assigned to the external validation set. In addition, to account for environmental variation in seed composition, the seed samples chosen for the calibration and external validation sets were derived from the three cultures (C1, C2, and C3). The prediction quality of NIRS analyses was then quantified by the SEP and the determination coefficient ($r_{V}^{2}$) between concentrations obtained from NIRS and from reference analysis for the validation set. The RPD was calculated as the ratio between the SD of the reference values and the SEP. RPD is indicative of the usefulness of the NIRS calibrations.

### Analysis of Seed Oil Content (Reference Method)

Oil was extracted following the standard NF V03-908 protocol (extraction by hot solvent with a “Soxlhet” extractor). About 1 g of seed was dried (103°C during 20 h) and ground in hexane with a grinder. Oil was then extracted with hexane by the Soxhlet method. The total seed oil content was expressed as percentage of the dried seed weight.

### Analysis of Seed Protein Content (Reference Method)

Phenol extraction of seed protein was adapted from Meyer et al. (1988). Ten mg of seeds were homogenized in a 2 ml tube containing a ceramic bead and 1 ml of an emulsion of 50% (v/v) phenol (previously equilibrated in 1 M Tris HCl pH8) in 0.1 M Tris HCl pH8 1% SDS using a Fastprep-24 Instrument (MP-Biomedical, maximal intensity, twice 1 min). After centrifugation (13 000 *g*, 20 min), 200 μl of the phenolic phase was accurately delipidated twice with 500 μl of hexane. One hundred μl of the phenol phase was taken after centrifugation (13 000 *g*, 10 min) and the proteins were precipitated with five volumes of methanol containing 0.1 M ammonium acetate at -20°C overnight. The precipitate was collected by centrifugation and washed four times with methanol (-20°C) containing 0.1 M ammonium acetate, and twice with 80% acetone (in water). The resulting pellets were dried under reduced pressure and then resuspended in 1 ml of 0.1 M Tris-HCl pH8 1% SDS. After overnight agitation, the fully dissolved solution was then cleared by centrifugation (13 000 *g*, 10 min) and the protein concentration was determined by spectrometry at 280 nm, assuming that 1 OD corresponds to 1mg/ml protein solution.

### Seed Nitrogen and Carbon Content (Reference Method)

Five mg of seeds, dried overnight at 100°C, were weighed on a lab balance model M2P (Sartorius, Göttingen, Germany) with a readability of 0.001 mg, then analyzed for nitrogen and carbon concentration by the Dumas combustion method (Anonymous, 1990) with an automated CN analyzer (Heraeus CN-Rapid, Hanau, Germany).

### QTL Detection

For each RIL, the mean value from three plants was taken for each measured trait for QTL analysis.

Quantitative trait loci analyses were performed using R/qtl library in the R environment (Broman et al., 2003; Arends et al., 2010) with standard methods for interval mapping (IM) and multiple QTL mapping (MQM) (Arends et al., 2010). First, IM was carried out to determine putative QTL involved in the variation of the trait, and then MQM model was performed on the same data: the closest marker to each local logarithm-of-odds (LOD) score peak (putative QTL) was used as a cofactor to control the genetic background while testing at another genomic position. The significance threshold (*p* < 0.05) of LOD was determined by permutation test (*n* = 1000) for each trait (Churchill and Doerge, 1994). The estimated additive effect (representing the mean effect of the replacement of the *Col-0* alleles by *Ct-1* alleles at the locus) and the percentage of variance explained by each QTL (R^2^) affecting a trait were obtained for the final MQM model.

## Author Contributions

PG, PB-M, and SJ established NIRS models. PG and SJ performed the statistical analysis and wrote the manuscript. AL and MD carried out the plant cultures, seed protein content (reference method) measurements and NIR spectrum acquisitions and predictions. PG and AL performed seed carbon and nitrogen content analysis (reference method). SJ performed the QTL detection experiments. All authors read and approved the final manuscript.

## Conflict of Interest Statement

The authors declare that the research was conducted in the absence of any commercial or financial relationships that could be construed as a potential conflict of interest.

## Footnotes

1 http://publiclines.versailles.inra.fr/

## Abbreviations

QTL: quantitative trait loci
RIL: recombinant inbred line
